# The learning curve of single‐port extraperitoneal robotic radical prostatectomy: Initial experience and outcomes from a newly graduated fellowship‐trained robotic surgeon

**DOI:** 10.1002/bco2.70184

**Published:** 2026-03-10

**Authors:** Jacob S. Hershenhouse, Simon Kim, Rafael Gevorkyan, Brian Hom, Michael Eppler, Patrick Ford, Ram Pathak, Riccardo Autorino, Andre Abreu, Ketan K. Badani, Simone Crivellaro, Sij Hemal

**Affiliations:** ^1^ Catherine and Joseph Aresty Department of Urology USC Institute of Urology, Keck School of Medicine Los Angeles California USA; ^2^ Department of Urology Mayo Clinic Jacksonville Florida USA; ^3^ Glickman Urological Institute Cleveland Clinic Cleveland Ohio USA; ^4^ Department of Urology Icahn School of Medicine at Mount Sinai New York New York USA; ^5^ Department of Urology University of Illinois at Chicago Chicago Illinois USA

**Keywords:** learning curve, minimally invasive surgery, radical prostatectomy, robotic surgery, single‐port surgery

## Abstract

**Introduction and Objective:**

This study aims to evaluate the learning curve and perioperative outcomes of single‐port extraperitoneal robotic radical prostatectomy (SP‐EP‐RARP) performed by a single surgeon at a high‐volume academic institution.

**Methods:**

A retrospective review of a prospectively maintained database was conducted for patients who underwent SP‐EP‐RARP from September 2023 to August 2025. Key metrics included operative time, estimated blood loss, conversion rate, nerve‐sparing status, pathological margin status and continence (0–1 pad for safety), and 30‐day complication rate. Descriptive statistics were used, and outcomes were analysed chronologically to assess for trends suggesting a learning curve.

**Results:**

The cohort included 53 patients who underwent extraperitoneal SP‐EP‐RARP. Median operative time was 213 min (IQR: 145–281). Median estimated blood loss was 100 mL (IQR: 75–125). Nerve‐sparing was attempted in 75.5% of cases. No assist *or plus* one ports were utilized. Final pathology revealed pT2 disease in 64.1% (34 cases), pT3a in 32.1% (17 cases) and pT3b in 3.7% (2 cases). Overall positive margin rate was 26.4%. Thirty‐day Clavien–Dindo Grades I–II complication rates were 11.3%, and no major complications or 90‐day complications were reported. Continence rates at 6 weeks, 3 months and 6 months were 72%, 72% and 75%, respectively. Stabilization of operative times and intraoperative outcomes occurred after approximately 25–30 cases.

**Conclusion:**

SP‐EP‐RARP can be safely implemented by a newly graduated fellowship‐trained robotic surgeon with acceptable oncologic and functional outcomes. Initial learning can be estimated at approximately 25–30 cases.

## 1 INTRODUCTION

The da Vinci Single‐Port (SP) robotic system (Intuitive Surgical, Sunnyvale, CA, USA), approved by the FDA in 2018, represents a paradigm shift in minimally invasive urological surgery.^1^ Unlike conventional multiport robotic systems, the SP platform utilizes a single incision with articulating instruments that provide enhanced manoeuvrability in confined anatomical spaces. This technological advancement promises reduced patient morbidity, less pain, expedited recovery, improved cosmesis and potentially shorter hospital stays.^2^ The initial SP adoption period represents a discovery phase, during which a restricted number of experienced surgeons have attempted to optimize this new technology, with urology being the first specialty to adopt this platform.

Single‐port robot‐assisted laparoscopic prostatectomy (SP‐RARP) was pioneered by Kaouk et al. who first reported the feasibility of this approach in 2019.^3^ Since then, various surgical approaches have been described, including both transperitoneal and extraperitoneal techniques. The extraperitoneal approach was championed by several groups that recognized its potential advantages attributed to the regionalization of the surgery to the target organ. These advantages include reduced risk of bowel injury, elimination of Trendelenburg positioning requirement, higher rates of same‐day discharge, decreased postoperative pain and preservation of the natural tissue planes that may facilitate anatomical reconstruction.^4^, ^5^, ^6^ This approach builds upon the well‐established principles of performing an extraperitoneal nerve‐sparing radical prostatectomy while incorporating the technological advantages of the single‐port platform.

However, despite promising improved ergonomics by decreasing instrument crowding and clashing in confined, small spaces, the transition from multiport to single‐port robotic surgery presents unique challenges even for experienced robotic surgeons. The altered ergonomics, instrument handling and degrees of freedom require a dedicated learning period. The SP platform introduces novel complexities including weaker tissue traction secondary to the more delicately designed instrumentation, altered visualization angles and modified triangulation techniques that differ significantly from conventional multiport approaches.^7^ Although the new features of the SP platform allow for the implementation of novel surgical strategies such as rapid adoption of extraperitoneal pelvic surgery and retroperitoneal renal surgery, they have added a certain level of complexity to procedures that were previously well‐standardized.

Understanding the learning curve is essential for establishing training protocols, ensuring patient safety and optimizing surgical outcomes. In surgery, the learning curve represents the relationship between a surgeon's experience and patient outcomes and estimates the number of patients at risk of suboptimal oncologic and functional outcomes associated with the learning process.^8^ Previous studies have demonstrated that the learning curve for conventional multiport robot‐assisted radical prostatectomy (MP‐RRP) ranges from 30 to 60 cases for technical proficiency, with variations depending on the specific outcomes measured and surgeon experience.^9^ However, the learning curve for SP‐RRP may differ significantly due to the unique characteristics of the single‐port platform.

The objective of this study was to evaluate the learning curve and perioperative outcomes of single‐port extraperitoneal robotic radical prostatectomy (SP‐EP‐RARP) performed by a single, recently graduated, fellowship‐trained robotic surgeon at a single, large academic medical institution, with a focus on identifying the thresholds at which postoperative functional and oncologic outcomes remain clinically acceptable, while simultaneously optimizing surgical efficiency. By analysing both technical and clinical parameters throughout the early adoption period, we aimed to provide insights that could inform training protocols and help guide surgeons considering implementating this novel approach.

## 2 METHODS

### 2.1 Study population

We included the first 53 consecutive patients undergoing SP‐EP‐RARP performed by a single newly graduated fellowship‐trained robotic surgeon (S.H.) at a high‐volume academic medical centre between September 2023 and August 2025. Starting September 2023 captures the surgeon's first independently performed single‐port extraperitoneal radical prostatectomies as the goal of this study is to present the surgeon's initial experience with the SP platform. Baseline demographics, clinical characteristics, perioperative variables and postoperative outcomes were collected in a prospectively maintained database and analysed retrospectively.

All patients underwent preoperative evaluation including prostate‐specific antigen (PSA) measurement, magnetic resonance imaging and tissue biopsy confirmation of prostate adenocarcinoma. Risk stratification was performed according to National Comprehensive Cancer Network (NCCN) guidelines^10^.

Beginning in Q3 2024 (case 28 onwards), a modified Hood approach was selectively adopted for appropriate candidates. Patient selection for the modified Hood technique was based on favourable oncologic characteristics (primarily NCCN low and favourable intermediate‐risk disease), lower preoperative Sexual Health Inventory for Men (SHIM) scores where preservation of erectile function was less critical and anatomy favourable for this approach. The standard anterior approach was maintained for patients with higher risk features, suspicion of extraprostatic extension or anatomy where oncologic control took precedence.

### 2.2 Study variables and outcome measures

#### 2.2.1 Baseline characteristics

Baseline patient characteristics included age at time of surgery, race, ethnicity, body mass index (BMI), Charlson Comorbidity Index (CCI), American Society of Anesthesiologists (ASA) score and relevant comorbidities including hypertension, diabetes mellitus and hyperlipidaemia. Clinical staging was performed according to the American Joint Committee on Cancer TNM classification system. Preoperative variables included PSA level, prostate volume, biopsy Gleason score, International Society of Urological Pathology (ISUP) grade, NCCN risk stratification, Briganti nomogram prediction score, baseline continence status, potency status, Sexual Health Inventory for Men (SHIM) score, International Prostate Symptom Score (IPSS) and quality of life (QOL) score.

#### 2.2.2 Intraoperative outcomes

Perioperative variables included console time (defined as time from robot docking to undocking), total operative time (defined as time from incision to closure), estimated blood loss (EBL), lymph node dissection performance, use of nerve‐sparing versus non‐nerve‐sparing, conversion to multiport or open surgery, additional port placement, drain placement and intraoperative complications classified according to the Clavien–Dindo system.

#### 2.2.3 Pathological outcomes

Pathological variables included specimen weight, pathological T stage (pT), nodal status (pN), ISUP grade, surgical margin status, margin extent (when positive) and the presence of extraprostatic extension. Positive surgical margins were defined as cancer cells touching the inked margin on final pathological examination.

#### 2.2.4 Postoperative outcomes

Short‐term postoperative outcomes included length of stay, postoperative hemoglobin levels, transfusion requirements, 30‐day and 90‐day complications according to Clavien–Dindo classification and 30‐day readmission rates. Functional outcomes were assessed at 6 weeks, 3 months, 6 months and 12 months postoperatively and included return of continence status (defined as being pad‐free or using a single pad for safety), IPSS and QOL scores. Biochemical recurrence was defined as two consecutive postoperative PSA values ≥0.2 ng/mL.

### 2.3 Statistical analysis

Descriptive statistics were used to summarize baseline characteristics, perioperative outcomes and postoperative results. Continuous variables are presented as median with interquartile range (IQR) or mean with standard deviation as appropriate. Categorical variables are presented as frequencies and percentages.

To assess the learning curve, outcomes were analysed chronologically according to case sequence and divided into annual quartiles. One‐way nonparametric ANOVA testing was employed to analyse differences in mean intraoperative outcomes over time. Statistical significance was defined as *p* < 0.05. All statistical analyses were performed using R software (R Foundation for Statistical Computing, Vienna, Austria). This study was approved by the University of Southern California institutional review board (HS‐012030‐CR021).

## 3 RESULTS

The study cohort included 53 consecutive patients who underwent SP‐EP‐RARP between September 2023 and August 2025. See Table 1 for baseline demographics.

**TABLE 1:** Demographic and preoperative characteristics.

Variables	Overall SP‐EP RARP (*n* = 53)	Learning phase (*n* = 25)	Maintenance phase (*n* = 28)
Age (year), median (IQR)	66.4 (60.5, 71.8)	65.8 (57.0, 72.5)	66.5 (59.1, 73.9)
BMI (kg/m^2^), median (IQR)	28.0 (24.3, 30.3)	27.6 (23.2, 32.0)	28.1 (22.8, 33.3)
CCI, mean	4.18	4.68	3.73
ASA physical status grading, mean	2.3	2.36	2.18
History of hypertension, *n* (%)	32 (60.4)	13 (52.0)	19 (67.9)
History of type 2 diabetes mellitus, *n* (%)	10 (18.9)	4 (16.0)	6 (21.4)
History of hyperlipidaemia, *n* (%)	25 (47.2)	14 (56.0)	11 (39.3)
History of prior pelvic surgery, *n* (%)	5 (9.4)	4 (16.0)	1 (3.6)
History of prior abdominal surgery, *n* (%)	16 (30.2)	10 (40.0)	6 (21.4)
Pre‐op Hgb, median (IQR)	13.1 (12.1, 14)	13.0 (11.1, 14.9)	13.2 (11.9, 14.8)
Pre‐op PSA (ng/mL), median (IQR)	7.2 (4.8, 8.4)	7.3 (4.5, 9.1)	7.2 (4.0, 10.4)
Prostate volume (mL), median (IQR)	38.3 (28.0, 46.3)	38.1 (27.4, 47.5)	43.9 (29.5, 58.3)
SHIM, mean (SD)	13.3 (9.7)	12.4 (8.8)	14.9 (9.4)
IPSS, mean (SD)	12.2 (10.4)	12.6 (7.6)	11.3 (8.9)
QoL, mean (SD)	2.7 (1.8)	2.6 (1.1)	3.2 (1.8)
Clinical T stage, *n* (%)			
T1	16 (30.2)	6 (24.0)	10 (35.7)
T2	30 (56.7)	17 (68.0)	13 (46.4)
T3	7 (13.2)	2 (8.0)	5 (17.9)
Biopsy Gleason score, *n* (%)			
3 + 3	10 (18.9)	4 (35.3)	6 (21.4)
3 + 4	25 (47.2)	12 (47.1)	13 (46.4)
4 + 3	9 (17.0)	6 (17.6)	3 (10.7)
4 + 4	4 (7.5)	1 (4.0)	3 (10.7)
>4 + 4	4 (7.5)	2 (8.0)	2 (7.1)
Unknown	1 (1.9)	0 (0)	1 (3.6)
Risk Assessment, *n* (%)			
Low	6 (11.3)	4 (17.6)	2 (7.1)
Intermediate	38 (71.7)	20 (82.3)	18 (64.3)
Favourable	20 (37.7)	11 (41.2)	9 (32.1)
Unfavourable	18 (34.0)	9 (41.2)	9 (32.1)
High	8 (15.1)	3 (0)	5 (17.9)
Very high	0 (0)	0 (0)	0 (0)
Metastatic	0 (0)	0 (0)	0 (0)

Abbreviations: ASA, American Society of Anesthesiologists; BMI, body mass index; CCI, Charlson Comorbidity Index; Hgb, haemoglobin; IPSS, International Prostate Symptom Score; IQR, interquartile range; PSA, prostate‐specific antigen; QoL, quality of life; SHIM, Sexual Health Inventory for Men.

The median operative time was 213 min (IQR: 145–281). The median estimated blood loss was 100 mL (IQR: 75–125). All but one procedure was successfully completed using the single‐port extraperitoneal approach; one case was converted to multiport due to the presence of extensive adhesions in the extraperitoneal space and significant pelvic lipomatosis. Nerve‐sparing technique was employed in 75.5% (40/53) of cases. No extra port or surgical drain was placed in our cohort. No intraoperative complications were observed in this series. No patients required intraoperative or perioperative blood transfusion.

Kruskal–Wallis analysis revealed significant temporal variations in key operative parameters across study quartiles. Operative time significantly decreased over time (*χ* ^2^ = 15.7, df = 6, *p* = 0.016), with mean operative times declining from 295.0 ± 24.0 min in Q4 2023 to 197.6 ± 36.1 min in Q2 2025, representing a 32% reduction.

**FIGURE 1:**
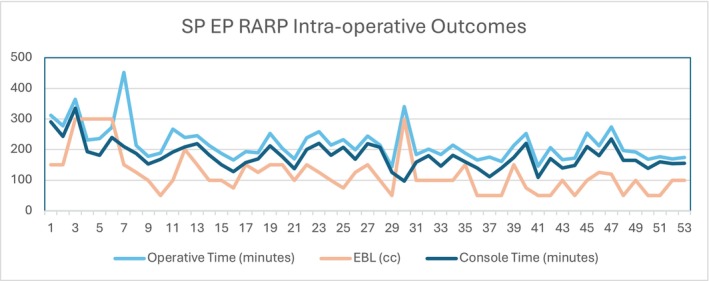
Intraoperative outcomes over time legend: blue: 6‐week continence; Orange: 3‐month continence; green: 6‐month continence.

Estimated blood loss also showed significant temporal improvement (*χ* ^2^ = 15.6, df = 6, *p* = 0.016). Mean blood loss decreased from 150.0 ± 0.0 mL in Q4 2023 to 83.5 ± 37.1 mL in Q2 2025, representing a 44% reduction, with notable reductions observed after the initial learning period. See Figure 1 for intraoperative outcomes over time.

Final pathological examination revealed pT2 disease in 64.1% (34/53) of cases, pT3a disease in 32.1% (17/53) of cases and pT3b disease in 3.8% (2/53) of cases. The overall positive surgical margin rate was 26.4% (14/53). Of 14 cases with positive margins, the majority (64.3%) were focally positive (<3 mm in size), representing 17% (9/53) of the total cohort. The majority of these positive margins showed Pattern 3 pathology at the margin (12/14; 85.7%) and 2/14 (14.3%) were Pattern 4. Positive margin rate was 20.6% (7/34) with pT2 disease and 36.8% (7/19) with pT3 disease. See Table 2 for pathological outcomes.

**TABLE 2:** Intraoperative outcomes by quartile.

	Quartile	*N*	Mean	SD	SE
Operative time	Q4 2023	2	295.000	24.042	17.0000
	Q1 2024	4	308.000	87.365	43.6826
	Q2 2024	8	256.875	86.012	30.4100
	Q3 2024	8	202.500	30.725	10.8628
	Q4 2024	3	235.000	21.656	12.5033
	Q1 2025	8	213.875	58.396	20.6462
	Q2 2025	17	197.588	36.191	8.7775
Estimated blood loss	Q4 2023	2	150.000	0.000	0.0000
	Q1 2024	4	250.000	100.000	50.0000
	Q2 2024	8	146.875	76.108	26.9082
	Q3 2024	8	118.750	29.124	10.2969
	Q4 2024	3	100.000	25.000	14.4338
	Q1 2025	8	128.125	74.926	26.4902
	Q2 2025	17	83.529	37.113	9.0013
Positive margins	Q4 2023	2	0.500	0.707	0.5000
	Q1 2024	4	0.750	0.500	0.2500
	Q2 2024	8	0.125	0.354	0.1250
	Q3 2024	8	0.125	0.354	0.1250
	Q4 2024	3	0.667	0.577	0.3333
	Q1 2025	8	0.625	0.518	0.1830
	Q2 2025	17	0.176	0.393	0.0953
Margin extent	Q4 2023	2	1.000	1.414	1.0000
	Q1 2024	4	1.000	0.816	0.4082
	Q2 2024	8	0.375	1.061	0.3750
	Q3 2024	8	0.125	0.354	0.1250
	Q4 2024	3	1.333	1.155	0.6667
	Q1 2025	8	1.000	1.069	0.3780
	Q2 2025	17	0.176	0.393	0.0953

**FIGURE 2:**
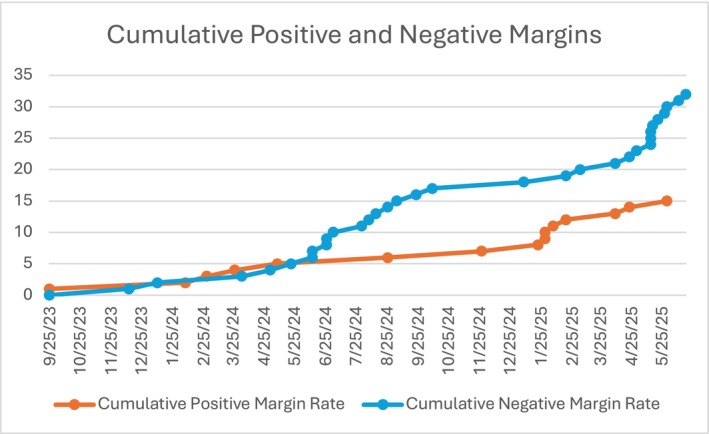
Pathological margins over time.

Positive surgical margin rates showed a trend toward improvement over time (*χ* ^2^ = 12.9, df = 6, *p* = 0.044). See Figure 2 for surgical margin rates over time. The proportion of cases with positive margins was highest in early quarters (50%–75%) and decreased to 20% in Q2 2025. Margin extent, when positive, also demonstrated a trend toward improvement (*χ* ^2^ = 13.5, df = 6, *p* = 0.035).

The 30‐day complication rate was 9.4% (5/53), with all complications classified as Clavien–Dindo Grade I–II. No major complications (Grade III or higher) occurred within 30 days of surgery. Complications included two cases of extraperitoneal urine leak managed with Foley catheter placement, one episode of acute urinary retention, one case of meatal stenosis that was treated with simple Foley catheter placement and one admission for severe bladder spasms. The 30‐day readmission rate was 1.9% (1/53) for the above case. Thus far, no evidence of biochemical recurrence has been found in any patient of this cohort. See Table 3 for postoperative outcomes.

**TABLE 3:** Intraoperative outcomes by quartile one‐way nonparametric ANOVA (Kruskal–Wallis).

	*χ* ^2^	df	*p*
Operative time	15.7	6	0.016
Estimated blood loss	15.6	6	0.016
Positive margins	12.9	6	0.044
Margin extent	13.5	6	0.035

**FIGURE 3:**
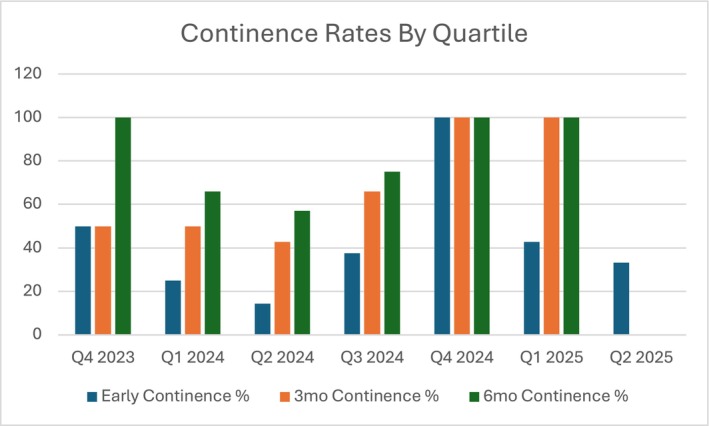
Continence rates by quartile.

Continence outcomes demonstrated variability across quarters with notable improvements in the later experiences of the surgeon. See Figure 3 for continence rates by quartile. Early continence rates (6 weeks) ranged from 14.3% to 100% across quarters, with the highest rates observed in Q4 2024 (100%), attributable to technique modification and use of the modified Hood technique. Three‐month continence rates showed progressive improvement from 42.9%–50% in the early quarters to 100% in Q4 2024 and Q1 2025. Six‐month continence rates demonstrated similar patterns, achieving 100% in both Q4 2023 and the later quarters (Q4 2024 and Q1 2025). Of 14 patients with at least 12 month follow‐up, 11 (79%) report full continence without the use of any pads. Three patients remained incontinent at 12 months with average pad use of 2 per day, managed with pelvic floor physical therapy; no patient in the cohort has gone on to require an adjunctive procedure such as an artificial urethral sphincter (AUS) or mid urethral sling (MUS).

Based on the temporal analysis, there were significant improvements in multiple perioperative outcomes of interest after approximately 25–30 cases, corresponding to the transition from Q3 2024 to Q4 2024. The early learning period (first 15–20 cases) was characterized by longer operative times (mean >250 min), higher blood loss (mean >140 mL) and elevated positive margin rates (>50%). The stabilization period (cases 25–30 onward) demonstrated consistently shorter operative times (mean <215 min), reduced blood loss (mean <130 mL) and improved margin rates (<30%).

## 4 DISCUSSION

This study demonstrates improvement in perioperative and postoperative outcomes of the SP‐EP‐RARP approach as a function of overcoming a single surgeon's learning curve. Our early experience with SP‐EP‐RARP demonstrates perioperative, functional and oncologic outcomes largely in line with those reported for contemporary multiport radical prostatectomy,^11^ with evidence of progressive improvement over time consistent with an adaptive learning phase.

The learning curve for SP‐RARP has been the subject of increasing investigation, with multiple studies providing valuable benchmarks for comparison. Our finding that operative time and complication rates stabilized after approximately 25–30 cases aligns well with several published series but differs from others, highlighting the variability in learning curve assessments.

Santarelli et al.^12^ analysed 119 extraperitoneal SP‐RARPs using CUSUM methodology and identified proficiency thresholds at 62 cases for SP‐RARP. Their approach, similar to ours, focused on extraperitoneal technique but utilized a more experienced surgeon (>10‐year MP‐RARP experience, >100 cases) compared to our early‐career surgeon. Despite the difference in baseline experience, their learning curve duration is longer than our findings, suggesting that the extraperitoneal approach may have inherent advantages for skill transferability.

Wu et al.^13^ employed CUSUM analysis for extraperitoneal single‐site SP‐RARP and found similar learning curves, reinforcing the consistency of findings across different institutions using extraperitoneal approaches. In contrast, Ramos‐Carpinteyro et al.^14^ evaluated transvesical SP‐RARP using CUSUM methodology on specific surgical steps (console, prostate excision, PLND and vesicourethral anastomosis) and identified plateaus within 10–30 cases, suggesting that the transvesical approach may have a steeper initial learning curve but potentially faster mastery of individual components.

Slusarenco et al.^15^ analysed learning curves in MP‐RARP and found that experienced surgeons typically required 40–60 cases to achieve consistent outcomes, while Zorn et al.^16^ reported learning curves of approximately 30 cases for fellowship‐trained laparoscopic surgeons transitioning to robotic surgery.

Positive surgical margin rates in our initial series (26.4% overall; 20.6% for pT2; 36.8% for pT3) are within the range reported in prior SP‐RARP series and approach established benchmarks for multiport RARP, where large series report 11%–28% overall positivity for organ‐confined disease.^17^ The temporal improvement in margin rates observed in our series aligns with findings from other learning curve studies, including those by Olivero et al.,^11^ who demonstrated that margin rates improve significantly after the initial learning phase in Retzius‐sparing approaches.

Several initial single‐port series consisting of both transvesical and extraperitoneal radical prostatectomies report positive margin rates of 20%–50%, to which our results are comparable.^11^, ^12^, ^13^, ^14^, ^15^, ^16^, ^17^, ^18^, ^19^ The consistency of these findings across different approaches and institutions suggests that SP‐RARP margin rates are acceptable during the learning period when appropriate case selection and surgical technique are employed.

Continence recovery reached 72% by 3 months and 75% by 6 months, which is comparable to early functional outcomes in high‐volume anterior and Retzius‐sparing multiport series.^17^, ^18^, ^20^, ^21^ While slightly below the best reported results exceeding 80%–90% at 1 year, our continence outcomes (79%) for patients with at least a 12‐month follow‐up are consistent with other learning curve series and demonstrate progressive improvement over time. In our current cohort nerve preservation was performed when oncologically feasible in patients with lower SHIM scores to promote return of continence. Sayyid et al.^22^ reported similar continence patterns during their learning curve for hood‐sparing techniques, with early continence rates improving significantly after technique modifications. Unfortunately, we do not have long term outcomes of continence when it comes to our more recent data due to lack of 12‐month follow‐up; however, of those with 1‐year follow‐up approximately 79% (11/14) were fully continent, and the remaining three patients, on average, use 2 pads per day and are content with undergoing pelvic floor physical therapy despite being offered adjunctive AUS or MUS placement by our reconstructive urology team.

The variability in continence outcomes across quarters in our series, with notable improvements in later experiences, underscores the importance of technique refinement during the learning period. The adoption of a modified Hood technique contributed to the achievement of 100% continence rates at 3 months in Q4 2024, demonstrating the potential for learning curve acceleration through evidence‐based technique modifications. The superior continence outcomes observed in Q4 2024 and beyond likely reflect both increased surgical experience and the benefits of the Retzius‐sparing technique for appropriately selected patients. The variable case volume across quarters, with substantially higher volumes in later periods, likely also contributed to accelerated skill consolidation. These factors should be considered when generalizing our findings to other practice settings.

Our findings have important implications for surgeons and institutions considering SP‐EP‐RARP adoption. The relatively short learning curve observed (25–30 cases) suggests that this approach can be safely adopted by appropriately trained surgeons with adequate institutional support. The ability to maintain acceptable oncologic outcomes during the learning period, as evidenced by stable margin rates and absence of major complications, supports the safety of SP‐EP‐RARP adoption when appropriate patient selection and surgeon preparation are ensured. This finding is consistent with reports from Pellegrino et al.,^23^ who demonstrated that learning curves for single‐port urological procedures can be successfully navigated without compromising patient safety when proper protocols are followed.

Our study has limitations that must be acknowledged when comparing with the broader learning curve literature. The single‐surgeon, single‐institution design limits generalizability, though it provides internal validity for learning curve assessment. The relatively small sample size compared to some multicentre studies may limit the power to detect smaller differences in outcomes over time.

The heterogeneity of outcome measures and learning curve definitions across studies makes direct comparisons challenging. While we focused on temporal trends in operative time and complication rates, other studies have used various CUSUM methodologies, specific procedural benchmarks or composite outcome measures. Future standardization of learning curve assessment methodologies would enhance the ability to compare findings across institutions and surgical approaches.

Despite these limitations, our experience contributes valuable data to the growing body of literature on SP‐RARP learning curves and supports the feasibility of this approach when undertaken with appropriate preparation and institutional commitment. This study differs from existing learning curve analyses that predominantly examine the transition of experienced multiport robotic surgeons to single‐port techniques. By evaluating clinical, functional and pathological outcomes of a surgeon starting their independent practice, this investigation offers valuable insights and benchmarks for resident and fellowship trainees preparing to establish their careers as robotic surgeons.

## 5 CONCLUSION

The continued evolution of robotic platforms and surgical techniques requires ongoing assessment of outcomes and refinement of approaches. Our experience contributes to the growing body of literature supporting the feasibility of single‐port approaches while highlighting areas for continued improvement and investigation. Overall, our findings suggest that SP‐EP‐RARP can be safely implemented by a newly graduated fellowship‐trained robotic surgeon who demonstrates acceptable early oncologic and functional outcomes. Initial learning can be estimated at approximately 25–30 cases. This learning curve is consistent with other published reports, and it emphasizes the importance of careful case selection during early adoption while demonstrating that acceptable outcomes can be achieved relatively rapidly with appropriate training and technique refinement.

## AUTHOR CONTRIBUTIONS

**Jacob S. Hershenhouse:** Conceptualization; data curation; formal analysis; investigation; methodology; writing—original draft; writing—review and editing. **Simon Kim:** Data curation; formal analysis; investigation; writing—review and editing. **Rafael Gevorkyan:** Data curation; investigation. **Brian Hom:** Data curation; investigation. **Michael Eppler:** writing—review and editing. **Patrick Ford:** Writing—review and editing. **Ram Pathak:** writing—review and editing. **Riccardo Autorino:** Writing—review and editing. **Andre Abreu:** Writing—review and editing. **Ketan K. Badani:** Writing—review and editing. **Simone Crivellaro:** Writing—review and editing. **Sij Hemal:** Conceptualization; methodology; project administration; supervision; writing—original draft; writing—review and editing.

## CONFLICT OF INTEREST STATEMENT

The authors declare no conflicts of interest.
